# Remarks from the Editor-in-Chief

**DOI:** 10.1017/psy.2025.10055

**Published:** 2025-11-05

**Authors:** Sandip Sinharay

**Affiliations:** ETS Research Institute

Dear *Psychometrika* Readers,

Welcome to the fourth Psychometrika issue of 2025. As a reminder, Psychometrika now publishes five issues per year—so there will be one more issue in 2025, to be published around December.

This Psychometrika issue starts with eight “Theory and Methods” section articles. In the first of these, Rongqian Sun, Xiangnan Feng, Chuchu Wang, and Xinyuan Song introduce the integration of Bayesian envelope methods into factor analysis models. The second, by Ying Liu and Steven Culpepper, presents new identifiability conditions for heterogeneous hidden Markov models. In the third article, Denis Federiakin and Mark Wilson propose a new bifactor model—the Completely Oblique Rasch Bifactor model—that allows for estimation of correlations between all dimensions. The fourth article, by Peida Zhan, Zhimou Wang, Gaohong Chu, and Haixin Qiao, suggests a teamwork cognitive diagnostic modeling framework. The fifth article, by Daniel Bengs, Ulf Brefeld, Ulf Kroehne, and Fabian Zehner, proposes two joint models for manual and automatic scores of automatically scored open-ended items. In the sixth article, Joseph Resch, Samuel Baugh, Hao Duan, James Tang, Matthew Madison, Michael Cotterell, and Minjeong Jeon propose an integrated Bayesian model for student progression in a longitudinal diagnostic classification modeling framework. The seventh article, by Youjin Lee and Youmi Suk, proposes a new evidence factors framework for fuzzy regression discontinuity designs with sequential treatment assignments, which may be influenced by different levels of decision-makers. In the final “Theory and Methods” article to appear in this issue, Youjin Sung, Youngjin Han, and Yang Liu extend the theory of generalized residuals, originally developed for models with categorical data, to encompass more general measurement models.

This Psychometrika issue then includes five articles from the “Application and Case Studies” section. In the first of these articles, Giuseppe Mignemi and Ioanna Manolopoulou suggest a flexible model under the Bayesian nonparametric framework for data involving multiple raters. The second article, by Hyeon-Ah Kang, introduces a refinement of the latent Markov model that relaxes the measurement invariance constraint on the test items. In the third of these articles, Jina Park, Ick Hoon Jin, and Minjeong Jeon propose a multi-state survival model to analyze action sequence data from log files, focusing on modeling test takers’ reaction times between actions. The fourth article, by Linh Nghiem, Jing Cao, Chrystyna Kouros, and Chul Moon, proposes a novel alignment approach that captures a wide range of temporal misalignment patterns in real-time emotional perception. In the final “Application and Case Studies” article to appear in this issue, Lijin Zhang, Benjamin Domingue, Leonie Vogelsmeier, and Esther Ulitzsch introduce and evaluate a model-based approach specifically designed to detect and account for careless respondents in visual analogue scales data.

The issue ends with a book review, written by Muwon Kwon, Nathan Quimpo, and Peter Steiner, of the 2025 book “Applied Causal Inference Powered by ML and AI” written by Victor Chernozhukov, Christian Hansen, Nathan Kallus, Martin Spindler, and Vasilis Syrgkanis.

Hope you enjoy the issue.

